# CD300a: An Innate Immune Checkpoint Shaping Tumor Immunity and Therapeutic Opportunity

**DOI:** 10.3390/cancers17111786

**Published:** 2025-05-27

**Authors:** Jei-Ming Peng, Hui-Ying Liu

**Affiliations:** 1 Institute for Translational Research in Biomedicine, Kaohsiung Chang Gung Memorial Hospital, Kaohsiung 83301, Taiwan; 2 Department of Urology, Kaohsiung Chang Gung Memorial Hospital, Graduate Institute of Clinical Medical Sciences, College of Medicine, Chang Gung University, Kaohsiung 83301, Taiwan

**Keywords:** CD300a, immune suppression, tumor immune evasion, myeloid cells, cancer immunotherapy

## Abstract

CD300 family members are immunoglobulin superfamily receptors that regulate immune cell function through either activating or inhibitory signals. Among them, CD300a is a prototypical inhibitory receptor, highly expressed in both myeloid and lymphoid lineages, and plays a pivotal role in the pathogenesis of inflammation and tumor immunity. CD300a transduces inhibitory signals in several immune cells—including mast cells, eosinophils, monocytes, dendritic cells (DCs), neutrophils, and natural killer (NK) cells—by recruiting SHP-1 phosphatase to immunoreceptor tyrosine-based inhibitory motifs (ITIMs) and suppressing activation pathways such as Toll-like receptor (TLR)-MyD88 and FcεRI signaling. Recent studies suggest that tumor cells may hijack CD300a-associated pathways to establish an immunosuppressive microenvironment that facilitates immune evasion, tumor survival, and potentially metastatic spread. Proposed mechanisms include reduced DC-mediated type I interferon (IFN) production, diminished NK cell cytotoxicity, and negative regulation of mast cell– and eosinophil-dependent anti-tumor responses. Although some of these findings are derived from in vivo models, the cumulative evidence positions CD300a as a critical immune checkpoint in tumor-associated immune regulation. In addition to its established roles in hematologic malignancies—including chronic lymphocytic leukemia, acute lymphoblastic leukemia, and acute myeloid leukemia—CD300a has also been implicated in modulating tumor-associated immune responses in other pathological contexts. While most studies emphasize its immune cell–mediated effects, emerging evidence suggests that CD300a may directly influence tumor progression by regulating immune homeostasis, intracellular signaling, and tumor microenvironment interactions. Collectively, these findings establish CD300a as a pleiotropic immunoregulatory molecule in both hematologic and non-hematologic malignancies, underscoring the need to further explore its broader relevance and therapeutic potential in cancer immunology.

## Simple Summary

The immune system maintains homeostasis by balancing activating and inhibitory signals. CD300a is an inhibitory receptor predominantly expressed on various immune cells, including mast cells, dendritic cells, monocytes, and NK cells. By recruiting phosphatases such as SHP-1, CD300a attenuates immune activation by suppressing key signaling pathways like TLR-MyD88 and FcεRI-mediated signaling. Recent studies suggest that tumors may exploit this inhibitory pathway to evade anti-tumor immune responses, for example by reducing type I interferon production or impairing NK cell cytotoxicity. This review highlights the immunoregulatory functions of CD300a and discusses its potential as a therapeutic target in cancer immunotherapy.

## 1. Introduction

The CD300 family comprises type I transmembrane molecules predominantly expressed in myeloid cells, which have multiple roles in regulating immune responses [1]. This defining property has served as the foundation for subsequent studies of their myriad activities in different immune settings. CD300 receptors participate in the regulation of allergic inflammation, with actions in mast cells, basophils, and eosinophils [2]. Of interest, some CD300 molecules can mediate activating and inhibitory immune responses, underscoring their functional diversity in immune modulation [3]. Furthermore, the CD300 family members participate in phagocytosis and cytokine production, which are crucial for immune homeostasis [4]. Taken together, the CD300 receptor family plays an important role in the regulation of immune responses and the development of inflammatory diseases.

### 1.1. Expression and Function of the Inhibitory Receptor CD300a

Based on the basic knowledge of the CD300 family, the role of one of its major inhibitory receptors, CD300a, has received increasing attention. CD300a (IRp60) is a type I transmembrane glycoprotein, predominantly expressed on myeloid cells, and is involved in down-regulating immune cell activation [2,5,6,7]. In addition, in eosinophils, CD300a cross-linking suppresses activation induced by IL-5, GM-CSF, and eotaxin, indicating a negative regulatory function [7]. Mechanistically, CD300a mediates negative signaling via its intracellular immunoreceptor tyrosine-based inhibitory motifs (ITIMs) [8]. It inhibits the cytotoxicity of natural killer (NK) cells in a ligand-dependent manner and induces apoptosis of activated T cells [9,10], highlighting its central position in immune suppression [11]. As a member of the immunoglobulin superfamily, CD300a is considered an important immunoreceptor for immune system regulation, suppressing inflammatory responses and abrogating overactivation of immune cells, making it a significant negative immune regulator [3].

### 1.2. CD300a: Genomic Localization, Structure, and Expression Regulation

CD300a is a member of the CD300 gene family, which consists of several closely related genes located on human chromosome 17 [1]. In humans, the CD300 multigene family is composed of seven unique members clustered in a 250 kb region of chromosome 17 [3]. Murine homologs of CD300a are members of the CMRF35-like family and are located on mouse chromosome 11 [3]. The genomic structure of human CD300 genes has been thoroughly investigated and the exact locations and orientations of individual genes have been determined. Such structural data can be helpful for functional comparison between human and murine CD300 family genes, which has been shown to be highly conserved through evolutionary analysis [12]. Additionally, CD300 gene expression has been reported to be cytokine- and environment-regulated, which reflecting their responsiveness to extrinsic signals [13]. This regulatory link is further supported by real-time PCR analyses demonstrating increased transcriptional activity under specific activating conditions [14,15] or response was observed. Although there are regulatory elements, the amount of CD300a expression appears to be largely constant in different experimental settings [16]. Nevertheless, some stimuli are reported to upregulate CD300a, suggesting a context-dependent regulation of its expression [17]. Moreover, recent studies indicate that Nlrp3 inflammasome activation affects CD300a gene expression, implicating innate immune signaling in the regulation of this receptor [18].

### 1.3. Distinct Roles of SHP-1 and SHP-2 in CD300a-Mediated Inhibition

CD300a-mediated immunosuppression mainly relies on protein tyrosine phosphatase SHP-1, but not on SHP-2 or SHIP. SHP-1 functions as a critical molecule in this signaling pathway. Functional analyses in DT40 B cells lacking SHP-2 and SHIP demonstrated that CD300a retains its inhibitory activity, suggesting that SHP-2 and SHIP are dispensable for this effect. However, in the absence of SHP-1 the inhibitory capacity of CD300a is severely impaired [19]. Moreover, treatment with SHP-1 inhibitor abrogates the CD300a-induced inhibitory effect, further emphasizing its critical role in this pathway [20]. This SHP-1 requirement has also been demonstrated in other cell systems; for example, it has been shown that depletion of SHP-1 in bone marrow-derived mast cells (BMMCs) markedly disrupts CD300a-mediated inhibition [21]. Therefore, SHP-1 acts as a critical molecular “switch” that unlocks the inhibitory signaling cascade downstream of CD300a.

### 1.4. Activating Members of the CD300 Family

In addition to the inhibitory receptor CD300a, several activating members have been identified in the CD300 family (Table 1). Of these, the expression of CD300b is restricted to neutrophils, macrophages, and mast cells, where it promotes activation and inflammation by interacting with the DNAX-activation protein of 12 kDa (DAP12) and therefore acts as an important activating immune regulator [22]. CD300c is abundantly expressed on human basophils and mast cells, where it functions to activate the cells and it is involved in allergic responses [23]. Moreover, CD300c protein is present on professional antigen-presenting cells (APCs) such as B cells, monocytes, macrophages, and dendritic cells (DCs) [24,25]. Similarly, CD300d is an activating receptor found mainly on monocytes and macrophages, extending the functional spectrum of the CD300 family [26]. Additionally, CD300e stimulates the pro-inflammatory cytokine production in monocytes and myeloid dendritic cells (mDCs), suggesting its potential role in the induction of the innate immune response [27,28]. However, the biological role of CD300e remains largely undefined and should be investigated further. One other family member, expressed primarily on bone marrow-derived cells such as monocytes, macrophages, DCs, granulocytes and mast cells is CD300f [12,29,30,31,32]. Similar to CD300a, CD300f has ITIM motifs in its cytoplasmic tail and recruits the inhibitory phosphatases to exerts suppressive effects [33]. CD300f is a negative regulator of mast cell activation [34,35] and inhibits neutrophils and monocytes in their antimicrobial functions [36,37]. It also binds to phosphatidylserine (PS) and regulates exocytosis in myeloid cells [38,39].

The immunoregulatory function of CD300 molecules is also induced via intracellular signals. CD300a-mediated inhibition is mediated by ITIM motifs located in its cytoplasmic tail, which upon ligation engage SHP-1 and SHP-2 phosphatases to transduce the inhibitory signals [8]. Notably, CD300f contains both activating and inhibitory motifs in its cytoplasmic tail, thus functioning as a dual regulative molecule during immune responses [33]. Thus, the activities of CD300a and CD300f are regulated by the balance between inhibitory signals produced by ITIMs and activating signals triggered by cross-linking to other receptors. CD300g lacks ITIM motifs and, unlike CD300a, is unable to recruit SHP-1 or SHP-2 via tyrosine residues in its cytoplasmic tail, nor does it signal via DAP12, as CD300b does. CD300g is expressed on high endothelial venules (HEVs) in lymph nodes, where it mediates lymphocyte rolling through its mucin-like domain, binding through its immunoglobulin (Ig) domain, and transendothelial migration in vitro [3,40,41,42]. Notably, although CD300c shows high homology to the inhibitory receptor CD300a, it signals through the activating machinery in human mast cells. Functional divergences between these CD300 molecules highlight the importance of the transmembrane domains of CD300 family members, which are required to interact with activating signaling partners [43] (Table 1).

**Table 1:** Expression and function of CD300 family receptors in leukocytes and vascular cells.

Receptor	Expressing Cells	Structural Features	Recruited Molecules	Functional Type	Notes	References
CD300a	Eosinophils, neutrophils, basophils, macrophages, and mast cells	Typical ITIM motifs	SHP-1, SHP-2	Inhibitory receptor	Binds phosphatidylserine (PS) and phosphatidylethanolamine (PE), negative regulation	[2,7,43,44,45,46]
CD300b	Neutrophils, macrophages, and mast cells	No ITIM, requires DAP12	DAP12 (activating adaptor)	Activating receptor	Enhances cells survival and activation	[22]
CD300c	Basophils, mast cells, B cells, monocytes, macrophages, and dendritic cells	No ITIM, requires DAP12	DAP12	Activating receptor	Enhances allergic responses and activation	[23,24,25]
CD300d	Monocytes and granulocytes	Activating type (prominent ITAM-like motif)	DAP12	Activating receptor	Participates in inflammation regulation	[26]
CD300e	Monocytes and myeloid dendritic cells	No ITIM, requires DAP12	DAP12	Activating receptor	Promotes production of inflammatory cytokines	[27]
CD300f	Monocytes, macrophages dendritic cells, mast cells and granulocytes	Multiple ITIM motifs (including non-classical ITIM)	SHP-1, SHP-2, PI3K (p85)	Inhibitory receptor	Promotes clearance of apoptotic cells, prevents autoimmunity	[12,29,30,31,32]
CD300g	Vascular endothelial cells	Mucin-like extracellular domain		Cell adhesion	Facilitates lymphocyte rolling and transmigration across the endothelium	[33]

## 2. Immunoregulatory Roles of CD300a Across Innate Immune Cell Subsets

### 2.1. Expression and Functional Regulation of CD300a in Monocytes and Macrophages

CD300a is expressed on a wide range of human leukocytes, including neutrophils, basophils, eosinophils, mast cells, monocytes, B lymphocytes, NK cells, and DCs, and is involved in the regulation of cellular proliferation, differentiation, apoptosis, and immune responses [5,47,48,49,50,51]. In mice, CD300a is also expressed at the cell surface of NK cells, macrophages, B cells, and mast cells, whereas another related molecule, CD300f, is expressed on neutrophils, DCs, mast cells, macrophages, and a subset of B cells, where it regulates cell activation, accumulation, migration, and secretory functions [20].

It has been demonstrated that there is a dynamic regulation of CD300a expression during monocyte differentiation. CD300a also exists on monocytes and its expression is reduced during differentiation of monocytes to monocyte-derived dendritic cells (MoDCs) [21]. Several inflammation- and stress-related stimuli such as LPS, IFN-γ, IFN-α, and hypoxia are able to regulate the expression of CD300a in monocytes and in DCs. Hypoxia-inducible factor 1 (HIF-1)–mediated upregulation of CD300a is reversed by HIF-1 inhibition, suggesting that CD300a may contribute to monocyte adaptation to inflammation and stress [3,52,53].

#### 2.1.1. Inhibitory Function of CD300a in TLR Signaling in Monocytes

**Figure 1:**
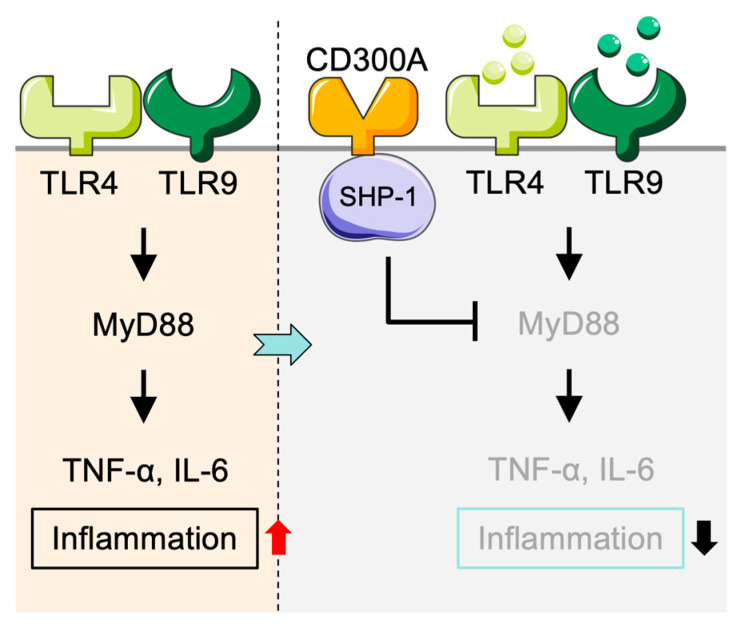
Negative regulation of inflammatory responses in monocytes/macrophages mediated by CD300A. TLR9 ligands and LPS stimulate the TLR9/TLR4-MyD88 cascade in monocytes and macrophages and produce a large amount of pro-inflammatory cytokines, including TNF-α and IL-6, to induce inflammation. Stimulation of CD300A recruits SHP-1 and subsequently suppresses MyD88 signaling, leading to decreased secretion of pro-inflammatory cytokines and thus an anti-inflammatory response. SHP-1: Src homology region 2 domain-containing phosphatase-1.

Immune and inflammatory pathways are regulated by Toll-like receptors (TLRs) in monocytes. CD300a suppresses monocyte activation by modulating TLR signaling pathways [20] (Figure 1). In particular, Kim et al. found that CD300a inhibits TLR4- and TLR9-induced pro-inflammatory responses in a human monocyte cell line, without any effect on TLR3-mediated signals [20]. Subsequent studies showed that CD300a inhibits SHP-1 activation, selectively attenuating MyD88 TLR signaling but not the TRIF-mediated pathway [1]. TRIF (TIR-domain-containing adapter-inducing interferon-β) is another adapter that operates in MyD88-independent manner for TLR3 and partly TLR4, and also contributes to antiviral immunity through IRF3 activation and type I interferon (e.g., IFN-β) production [54,55]. As CD300a does not interfere with TRIF-dependent signaling, this suggested its regulatory mode is also designed to inhibit inflammatory responses but not antiviral immunity. This selective suppression of inflammation may contribute to the pathogenesis of immune-related diseases.

#### 2.1.2. Effects of CD300a Deficiency or Knockdown on Function

Indeed, BMMCs and macrophages from CD300a^−/−^ mice produce higher levels of pro-inflammatory cytokines when stimulated by LPS in the presence of apoptotic cells, which further indicates that CD300a has an anti-inflammatory role in this situation [21]. Conversely, siRNA-mediated suppression of CD300a in macrophages increases phagocytosis, again supporting an inhibitory role for CD300a in the regulation of monocyte and macrophage function [8].

### 2.2. Expression and Function of CD300a in DCs

**Figure 2:**
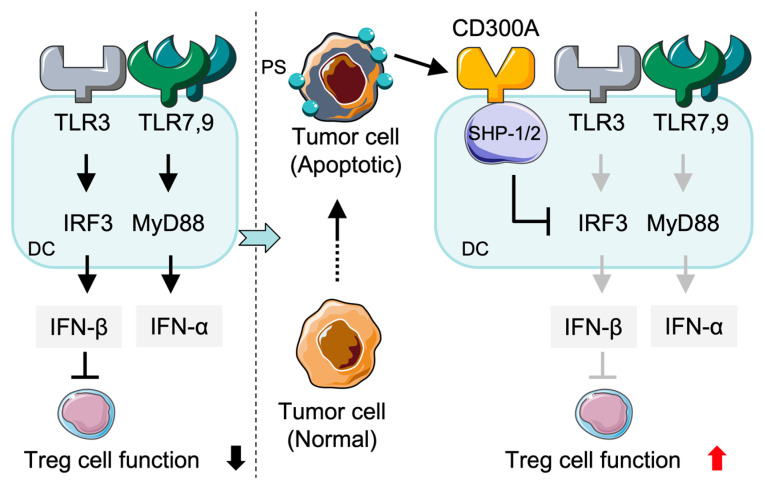
CD300A-mediated suppression of anti-tumor dendritic cell (DC) responses. In the TME, apoptotic tumor cells expose PS, which binds to CD300A on DCs. The TLR3- and TLR7/9-mediated IRF3 and MyD88 pathways were inhibited through the activation of SHP-1/2-phosphatases by CD300A engagement. This leads to reduced production of type I interferons (IFN-β and IFN-α) and increased Treg cell function, thereby promoting tumor immune evasion. PS: phosphatidylserine; SHP: Src homology region 2 domain-containing phosphatase.

CD300a is an inhibitory receptor expressed on human DCs and contributes significantly to regulating their activity by preventing excessive immune responses and maintaining immune homeostasis [13,46] (Figure 2). The suppressive role is required for prevention of immune dysregulation and for generation of balanced immune responses. However, recent findings have indicated that CD300a may not function exclusively as an inhibitory receptor; under certain immunological conditions, it may also exhibit activating properties [56]. This phenomenon of dual functions is mediated by the immune microenvironment and is context-dependent. Evidence for this dual role is provided by studies in plasmacytoid dendritic cells (pDCs). TLR7 ligand-induced IFN-α r production is significantly elevated in CD300a-deficient animals, which suggests a role for CD300a in the modulation of pDC cytokine production [57]. These results imply the involvement of CD300a in protection against excessive type I IFN and fine-tuning of pDC-mediated immune responses. In parallel, type I IFNs, especially IFN-β, have been shown to regulate T cell immunity through their effects on regulatory T cells (Tregs). Specifically, IFN-β signaling via the interferon-α/β receptor (IFNAR) in Tregs attenuates their suppressive function in both viral and tumor contexts. Treg-specific IFNAR-deficient mice exhibit heightened Treg activity, increased expression of activation markers (CD44, Ki-67, ICOS, PD-1), and reduced CD8⁺ T cell responses, ultimately resulting in impaired tumor control [58].

In addition to CD300a, other members of CD300 family can also regulate immune responses in different leukocyte populations. CD300c expression in pDCs, for instance, may enhance cytokine production following Toll-like receptor (TLR) activation [57]. Although CD300c lacks ITIM, it may exert compensatory or co-stimulatory function when co-expressed with inhibitory CD300a by regulating type I IFNs and TNF-α production after TLR7/9 stimulation. In addition, in intestinal systems of immunity CD300f-expressing DCs participate in the clearance of apoptotic cells, thereby promoting immune tolerance and maintaining intestinal homeostasis [59]. CD300f^−/−^ mice exhibit enhanced inflammatory reactions, indicating the critical role of CD300f in immune tolerance. Nevertheless, it is unknown whether CD300f is a functional counterpart of CD300a in controlling IFN-α production from DCs. In the tumor microenvironment (TME), PS-positive tumor EVs contribute to inhibition of TLR3-mediated IRF3 and IFN-β expression through binding with CD300a on DCs. As a consequence, more tumor-infiltrating Tregs are generated and anti-tumor immune response is suppressed (Figure 2). These results demonstrate that CD300a-mediated inhibition of TLR3 signaling results in inhibition of type I IFN production and formation of an immunosuppressive TME [60].

Similar mechanisms may be employed by other CD300 family members in order to “quickly” sense (e.g., the presence of bacterial pathogens in) the extracellular environment and thus activate/inhibit certain dendritic cell subsets in order to adjust the balance of immune responses [61]. It has been previously demonstrated that therapeutically relevant factors including GM-CSF and IL-4 which stimulate the differentiation of monocytes downregulate the expression of CD300f. After differentiation, re-stimulation with the TLR4 ligand LPS does not re-induce CD300f expression, suggesting that CD300f regulation is well-controlled throughout the monocyte–DC transition and potentially affects later immune responses [29].

### 2.3. CD300a-Mediated Inhibition of Mast Cell Activation in Allergic Responses

**Figure 3:**
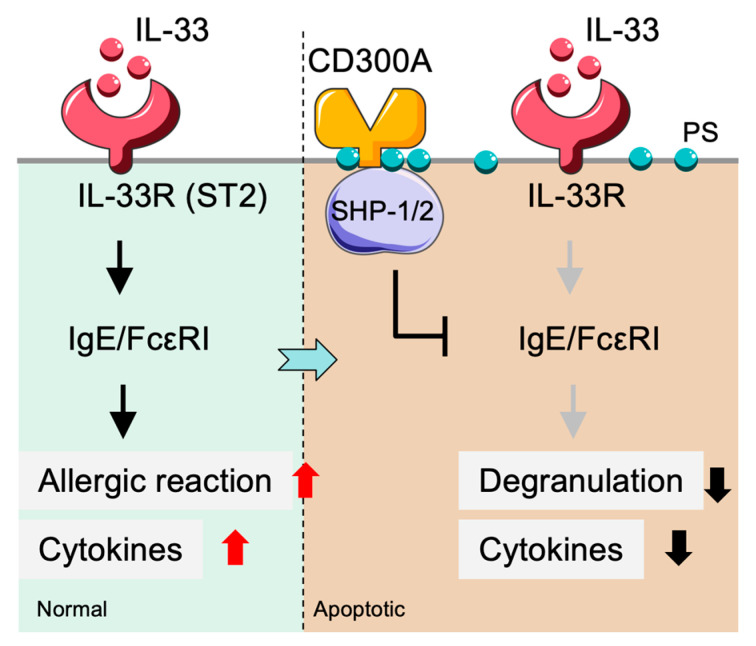
CD300A-mediated suppression of mast cell–driven allergic inflammation. Mast cells are activated by IL-33 via its receptor IL-33R (ST2), which promotes IgE/FcεRI-dependent allergic responses and cytokine release. Engagement of CD300A by apoptotic cells with externalized PS leads to recruitment of SHP-1/2 phosphatases, which inhibit FcεRI signaling. This leads to reduced degranulation and decreased cytokine secretion, thereby suppressing allergic inflammation. PS: phosphatidylserine; SHP: Src homology region 2 domain-containing phosphatase.

Mast cell activation is a critical component of allergic inflammation, which is tightly controlled by several major signaling cascades. Interleukin-33 (IL-33), a cytokine implicated in allergic responses and contact hypersensitivity, potentiates FcεRI-dependent mast cell activation. Nevertheless, this activation is markedly suppressed in the presence of CD300a, indicating that CD300a serves as an inhibitory checkpoint in the regulation of mast cell activity [62] (Figure 3). Beyond allergic contexts, tumor cells expressing PS can also engage CD300a-mediated processes to inhibit mast cell degranulation and the release of cytokines [63,64]. PS, which is normally confined to the inner leaflet of the plasmalemma of viable cells, is exposed on the outer leaflet during cellular stress, apoptosis, or tumorigenesis, providing an essential “eat-me” signal to the immune system [65]. This mechanism allows tumor cells to engage CD300a via PS exposure, thereby dampening immune responses, and implies that CD300a may also participate in tumor immune regulation in addition to its role in allergy. To elucidate this process further, a bispecific Fab fragment with binding affinity for both CD300a and PS was used, resulting in enhanced CD300a activation. This bispecific antibody potently suppressed mast cell degranulation via FcεRI and demonstrated the potential therapeutic usefulness of targeting CD300a on mast cells in regulating allergies and immunity [66]. Such dual-targeting approaches may offer novel strategies for regulating mast cell function in allergic disease and tumor-related immune suppression.

Beyond its role as a ligand for CD300a, externalized PS also acts as a broader immunoregulatory signal within the tumor microenvironment, where it modulates immune cell behavior and contributes to tumor immune evasion. PS exposure is not only a hallmark of apoptotic cells but is also observed in stressed yet viable tumor cells [67,68]. It plays active roles in promoting cell fusion (e.g., between tumor cells or tumor–macrophage hybrids) and driving M2-like macrophage polarization, thereby facilitating immune escape [69].

### 2.4. Immunoregulatory Role of CD300a in Eosinophils and Interaction with the TLR/MyD88 Pathway

**Figure 4:**
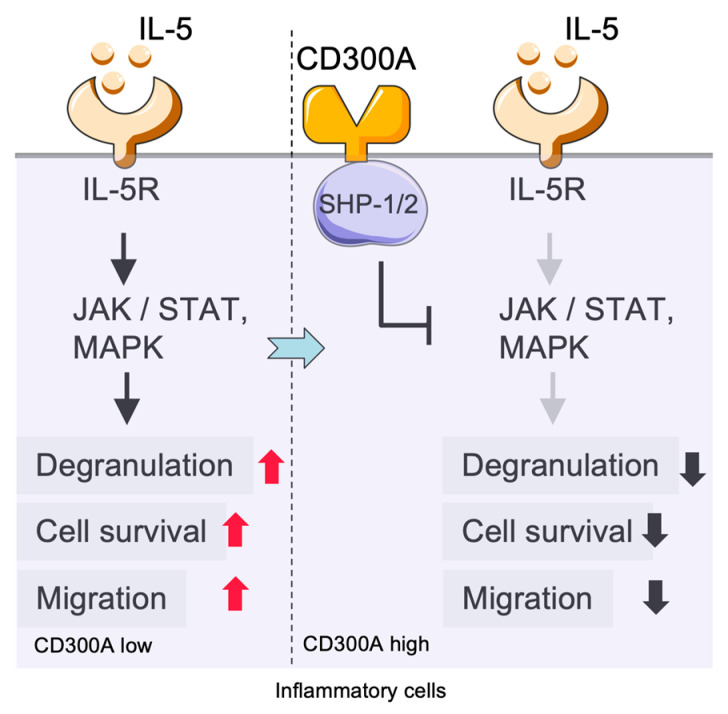
CD300A-mediated negative regulation of eosinophil activation and migration. IL-5 stimulation through IL-5R activates JAK/STAT and MAPK pathways in eosinophils, promoting degranulation, cell survival, and migration. High CD300A levels trigger SHP-1/2 phosphatases, which block distal signals transduction and most notably degranulation, cell survival, and migration. SHP: Src homology region 2 domain-containing phosphatase.

Eosinophils express multiple TLRs, and these cells can be activated with the MyD88 pathway, contributing to host defense against pathogens [45,70]. The direct link between eosinophils, the TLR/MyD88 pathway, and CD300a is not well known. CD300a, an inhibitory receptor expressed on eosinophils, serves to suppress eosinophil activation and downstream immune responses [71] (Figure 4). Using an allergic peritonitis model in mice, it was observed that injection of the antibodies blocked CD300a-mediated suppression of migration in the peritoneal cavity and resulted in an augmentation of eosinophil infiltration, suggesting that CD300a physiologically modulates the trafficking of eosinophils [72]. Moreover, CD300a cross-linking on human eosinophils suppressed IL-5-induced cell survival and degranulation, highlighting the inhibitory function of CD300a in the regulation of eosinophil responses [7].

Eosinophils also respond to innate immune stimuli in addition to CD300a-mediated regulation. Although TLR2 expression is not significantly elevated on eosinophils, TLR2-ligand stimulation leads to MyD88-dependent, cytokine production [2]. These results indicate that eosinophils can integrate multiple signals (e.g., from TLRs and CD300a) to finely modulate their immune responses [2].

### 2.5. CD300a in Neutrophils

**Figure 5:**
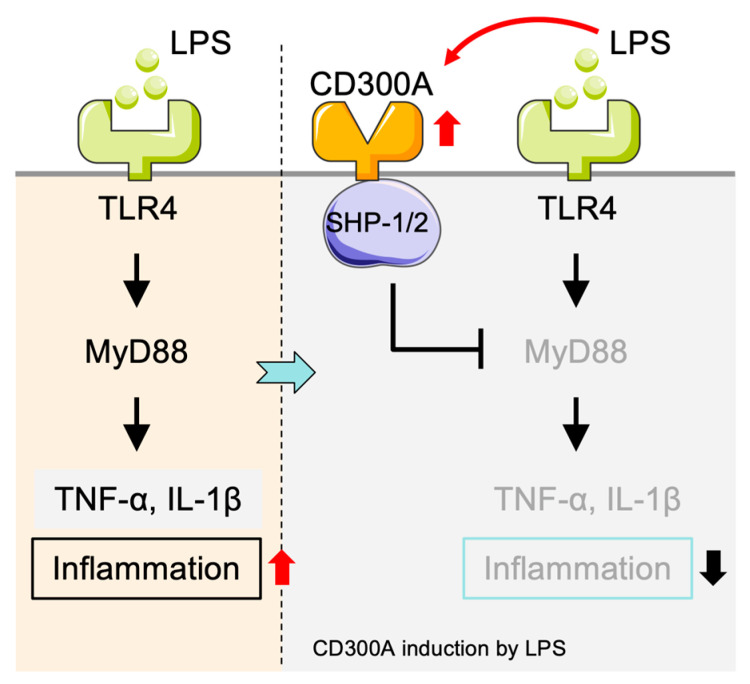
CD300A-mediated negative regulation of inflammatory responses in neutrophils. LPS-induced TLR4-MyD88 signaling in neutrophils leads to the production of pro-inflammatory cytokines including TNF-α and IL-1β, thereby inducing inflammation. LPS also induces the CD300A expression, which targets the phosphatases SHP-1/2 to suppressing TLR4-mediated signaling. This suppresses cytokine production and attenuates the inflammatory response. SHP: Src homology region 2 domain-containing phosphatase.

In neutrophils, stimulation with lipopolysaccharide (LPS) induces cellular activation and leads to a rapid upregulation of CD300a expression [61]. CD300a expression is dynamically regulated in inflammation, highlighting its important role in immunoregulation [3,47]. In the process of neutrophil activation, early induction of CD300a has been proposed to serve as a negative feedback mechanism, limiting excessive activation and helping to restore the balance between activating and inhibitory signaling pathways within the cell [61] (Figure 5).

### 2.6. Inhibitory Function of CD300a in NK Cells

NK cells are essential members of the innate immune system, and their cytotoxicity is tightly regulated by the balance between inhibitory and activating receptors [63]. NK cells express a diverse repertoire of receptors that collectively determine whether activating or inhibitory signals are triggered in response to the expression of ligands on the surface of target cells, and therefore respond accordingly [73]. This dynamic regulation is achieved through the interplay of inhibitory receptors (such as KIRs and NKG2A), which primarily recognize major histocompatibility complex class I (MHC I) molecules on healthy cells. When MHC I molecules are sufficiently expressed, NK cells remain quiescent. Infection, transformation, or cellular stress, in contrast, reduces MHC I expression and induces upregulation of activating ligands (e.g., MICA/B and ULBPs). This shift activates NK cells due to their receptors like NKG2D to mediate cytotoxicity. This process, known as “missing-self recognition”, is critical for NK cell identification of abnormal cells [74,75,76].

**Figure 6:**
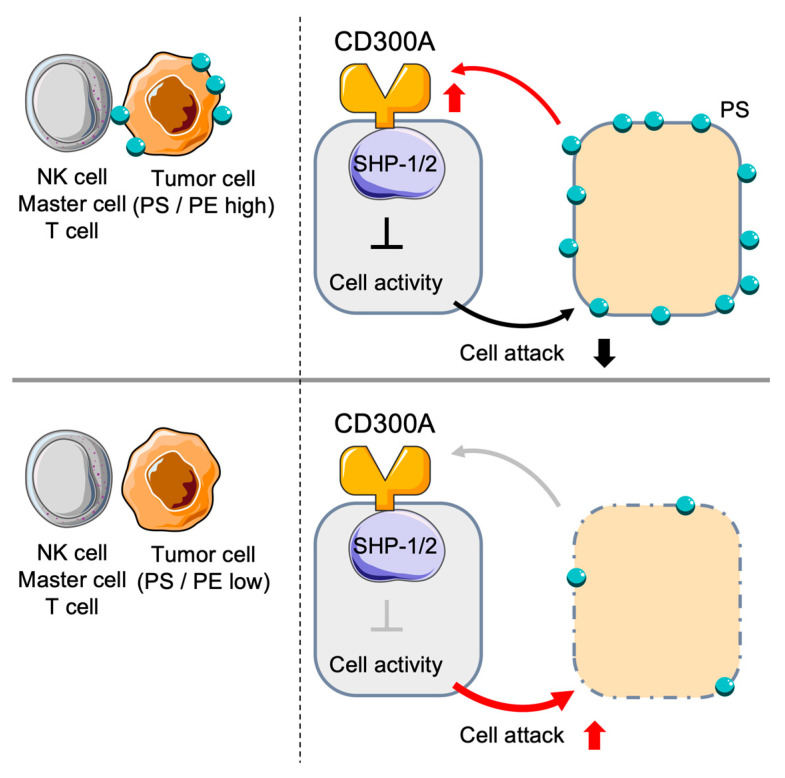
CD300A-mediated negative regulation of NK cell cytotoxicity. NK cells recognize tumor cells with high levels of PS or PE exposure, which engage CD300A and recruit SHP-1/2 phosphatases. CD300A stimulation potently inhibits NK cell cytotoxicity, permitting less tumor cell lysis. In contrast, the tumor cells expressing low levels of PS/PE fail to activate CD300A which leads to the increased activity of the NK cells. PS: phosphatidylserine; PE: phosphatidylethanolamine; SHP: Src homology region 2 domain-containing phosphatase.

Novel findings have shown that CD300a inhibits NK cytotoxicity through interaction with PS [63] (Figure 6). PS, which predominantly resides in the inner leaflet of the plasma membrane, becomes externalized during apoptosis or cellular stress. Blocking PS partially restores NK cell cytotoxic function, confirming the role of CD300a in inhibiting NK cell activity [63]. As an inhibitory receptor, CD300a interacts with apoptotic cells in a PS-dependent manner [77]. Upon phosphorylation of its ITIMs, CD300a associates with inhibitory phosphatases including SHP-1 and SHP-2, resulting in the inhibition of downstream signaling [46]. Immunoprecipitation studies have also demonstrated that cross-linking of CD300a efficiently recruits SHP-1 and SHP-2, consistent with its inhibitory signaling role [19]. Human NK cells treated with anti-CD300a antibodies show CD300a-dependent tyrosine phosphorylation and its association with SHP-1 and SHP-2 [46,78]. Functional inhibition of CD300a also interrupts SHP-mediated inhibitory signals, enhancing NK cell responses [46].

Specifically, members of the CD300 receptor family, particularly CD300a, contain ITIMs within their cytoplasmic domains, which play a critical role in immune regulation by recruiting the phosphatases SHP-1 and SHP-2. Upon phosphorylation, these ITIMs serve as docking sites for the SH2 domains of SHP-1 and SHP-2, and the phosphatases are recruited to the receptor complex. Once recruited, these phosphatases dephosphorylate key signaling molecules in close proximity, thereby effectively attenuating downstream immune activation signals [12]. As previously described, CD300a negatively regulates FcεRI-driven signaling in mast cells by recruiting SHP-1 in a phosphorylation-dependent manner through its ITIM motifs [3]. Similarly, CD300a regulates monocyte activation through SHP-1–mediated dephosphorylation and decreased production of proinflammatory cytokines [79].

## 3. CD300a in Tumor Immunology

### 3.1. CD300a-Mediated Signaling in Hematologic Malignancies

CD300a may contribute to tumor progression in hematologic malignancies, particularly in acute myeloid leukemia (AML). Sun et al. demonstrated that CD300a promotes upregulation of PECAM1 and ADCY7, thereby activating the AKT/mTOR signaling pathway, which may support AML cell survival and proliferation [80]. Although PECAM1 is traditionally recognized as an endothelial cell adhesion molecule and is not classically associated with intracellular signaling in tumor cells, its regulatory role in the AML context remains to be clarified. These findings suggest a potential role for CD300a in modulating oncogenic pathways in AML, although further validation is needed to confirm direct mechanistic links.

In diffuse large B-cell lymphoma (DLBCL), high CD300a expression correlates with poor prognosis and increased tumorigenicity. CD300a has been implicated in promoting tumor growth by modulating the PI3K-AKT signaling axis, particularly in early T-lineage lymphomas (ETTLs). Pharmacological inhibition or genetic deletion of CD300a reduces tumorigenicity both in vitro and in vivo models [49], suggesting its functional involvement in reshaping the immune landscape and promoting lymphomagenesis. However, current evidence remains limited, and further studies are required to validate these findings and elucidate the underlying mechanisms. In particular, it remains unclear whether CD300a exerts its tumor-promoting effects through direct action on malignant cells or indirectly by modulating immune cells such as monocytes and dendritic cells within the tumor microenvironment.

### 3.2. Possible Roles of CD300a in Solid Tumors

Although CD300a expression is primarily restricted to hematopoietic cells such as monocytes, macrophages, and dendritic cells, as confirmed by databases including the Human Protein Atlas, several studies have investigated its potential roles within the tumor immune microenvironment of solid tumors. In a breast cancer model, CD300a was shown to suppress mast cell–mediated anti-tumor responses by binding to PS and PE, which are enriched on tumor cell membranes [81]. In vivo studies demonstrated that CD300a-deficient mice exhibited smaller tumors, increased mast cell activation, and reduced IL-10 levels. While mast cells are not considered major effectors of anti-tumor immunity in most solid tumors, these observations suggest that CD300a may contribute to maintaining an immunosuppressive environment. However, it is likely that multiple immunological factors, including changes in cytokine profiles such as IL-10, contribute to these effects, and further investigation is necessary to establish causality.

In addition, the ligands of CD300a, PS and PE, are known to be present on the surface of tumor cells in gastric, ovarian, and melanoma cancers [82,83,84]. These observations imply that CD300a-expressing immune cells may interact with tumor cells via these ligands, potentially triggering inhibitory signals that suppress anti-tumor responses. Nevertheless, whether CD300a exerts a direct functional role in tumor progression or immune evasion in these solid tumors remains speculative and should be interpreted with caution.

## 4. Cross-Talk: Linking Immune Modulation to Tumor Progression

### 4.1. CD300a-Mediated Modulation in TME

Xu et al. reported that CD300a interacts with CD1C and CX3CR1, both of which are key mDCs markers that have immune regulatory activity in the TME of AML [85]. These interactions may significantly influence immune evasion and therapeutic efficacy in AML. CX3CR1⁺ DCs express high levels of PD-L1 (CD274) and PD-L2 (PDCD1LG2) in AML, which mediate T cell suppression and immunosuppressive TME formation [85]. In the presence of CX3CR1, co-expression of CD300a may further exacerbate T cell dysfunction and exhaustion. Indeed, CD1C⁺ DCs have been correlated with the induction of anti-tumor T cell responses through production of activating cytokines and expression of co-stimulatory molecules that support the function of both CD4⁺ and CD8⁺ T cells [86,87]. CD300a may impair this function by suppressing CD1C⁺ DC activity and consequently decrease antitumor immunity and promote immune evasion by AML cells.

#### 4.1.1. CD300a and TME

The TME consists of multiple immune cells, predominantly of myeloid origin, which via recruitment and polarization, support tumor outgrowth and immune evasion [88,89]. A large fraction of tumor-infiltrating immune cells expresses high levels of inhibitory receptors, including CD300a [46,85]. Myeloid-derived cells, particularly monocytes and macrophages, express inhibitory receptors at high levels, underscoring the pivotal role of CD300a in sustaining immunosuppression within the TME.

Tregs also play a key role in shaping the immunosuppressive TME. Tumor-derived extracellular vesicles carrying PS bind to CD300a on DCs, and suppress TLR3-mediated IFN-β production, leading to enhanced in vitro accumulation of tumor-infiltrating Tregs [60,90]. CD300a appears essential for the homeostasis of tumor-infiltrating cells, as profound changes in the phenotypic and functional distribution of immune cells in the TME have been identified in CD300a-deficient mouse strains, thus supporting a fundamental role of CD300a in the promotion of the tumor immune homeostasis [60]. Moreover, the transcription factor PPARβ/δ has been reported to elevate expression of CD300a, indicating the potential of fatty acid metabolism to regulate CD300a in the TME, although current evidence primarily originates from intestinal immunity studies [91].

#### 4.1.2. CD300a and Pathological Neutrophil Activation

Pathologically activated neutrophils, referred to as PMN-MDSCs, modulate the immune microenvironment in the TME and facilitate tumor dissemination [92]. In preclinical models, genetic deletion or functional inhibition of CD300a increase immune reactivity and limit tumor growth, further indicating its classification as an immune checkpoint [80]. The function of CD300a in the regulation of neutrophils has been relatively well defined, but that of CD300f has not been well studied, particularly in the context of neutrophil-mediated tumor immunity [1,3]. These findings highlight a role for CD300a in promoting tumor-associated immunosuppression and suggest its potential as a therapeutic target.

### 4.2. CD300a and NK Cells in Cancer

Elevated CD300a expression on NK cells has been demonstrated to reduce their cytotoxicity against tumor cells. Overexpression of CD300a in NK cells is associated with impaired anti-tumor activity, as demonstrated by decreased survival in xenograft mouse models, supporting the role of CD300a-mediated inhibition in tumor clearance by NK cells. Inhibition of CD300a interaction with PS by antibodies restores NK cell function, leading to the upregulation of proteins and cytokines associated with cytotoxicity, thereby enhancing NK effector responses. These findings indicate the repressive effect of CD300a on tumor immunity through NK cells [93].

Moreover, studies on CD300a/c expression in human CD4⁺ memory T cell subsets have demonstrated that CD300a/c⁺ CD4⁺ T cells are largely TH1 and produce IFN-γ which is essential for anti-tumor immunity. Conversely, CD300a/c^−^ CD4⁺ T cells are more related to a TH17 profile, expressing IL-17A, which may promote tumor progression in certain tumor microenvironments. Therefore, CD300a-mediated inhibition of TH1 activity may contribute to immune evasion by suppressing anti-tumor responses [93].

In addition to classical immune cells, CD300a is expressed on other cell types. Human umbilical cord blood-derived mast cells (MUCBMCs) express CD300a, which acts as a negative regulator of mast cell degranulation and cytokine production [6]. In NK cells, CD300a transduces inhibitory signals via its intracellular ITIM motifs by recruiting SHP-1 and SHP-2 phosphatases. Cross-linking of CD300a inhibits NK cell cytotoxicity and IFN-γ production and may therefore reduce the immune activation and suppressing overall immune responses [78].

### 4.3. CD300a and Mast Cells in Cancer

Mast cell activation is significantly increased in CD300a-deficient mice, resulting in heightened severity of allergic inflammation [21]. In asthma models, mast cells are key regulators of disease development and CD300a negatively regulates allergic responses by modulating mast cell activity [2,21]. Subsequent studies demonstrated that the direct inhibition of mast cell inflammation is due to the PS binding to CD300a. These results suggest that ligation of CD300a by its ligands induces inhibitory signaling to modulate various immune responses [46].

In non-small cell lung cancer (NSCLC), the expression of CD300a is elevated, and its high expression is negatively correlated with NSCLC tumor suppression, potentially through inhibition of the Wnt/β-catenin signaling pathway, thereby slowing tumor progression [51]. In chronic asthmatic models, CD300a-targeting bispecific antibodies have been shown to reverse airway inflammation and tissue remodeling [94]. In addition, artificial bispecific antibodies that target CD300a have been developed which are able to modulate the activity of mast cells and other immune cells, representing a promising therapeutic strategy for both inflammatory and oncologic disease [6,95].

## 5. Therapeutic Implications

### 5.1. CD300a Blockade as Monotherapy

Blockade of CD300a alone has demonstrated significant anti-tumor efficacy in preclinical breast cancer models, supporting its potential as a standalone immunotherapeutic strategy. Treatment with a blocking anti-CD300a antibody significantly suppressed tumor growth in a murine 4T1 breast cancer model, accompanied by robust activation of immune cells in the TME, such as mast cells and T cells. Importantly, this effect was independent of co-treatment with other immune checkpoint inhibitors, such as PD-1 blockade, confirming the intrinsic anti-tumor capability of CD300a inhibition [81].

CD300a, a myeloid lineage-expressed inhibitory IFN-γ-induced immune checkpoint receptor with ITIMs, compromises NK cell-mediated eradication of hematologic malignan-cies (HMs). Therefore, CD300a is an attractive target for extending NK cell-based immu-notherapy. PS was externalized on the surface of tumor cells, and CD300a bound specifi-cally to PS. Ectopic CD300a expression in NK cells markedly reduces their cytotoxic activ-ity on hematologic tumor cells and promotes the in vivo progression of the tumor. In con-trast, the blockade of the CD300a-PS interaction by antibodies increases the transcription of cytotoxic proteins and cytokines in the NK cells, restoring their tumor-killing function. Clinical evidence also indicates that upregulated CD300a expression is associated with NK cell exhaustion and correlates with poor prognosis in patients with both hematologic and solid malignancies [93]. In the 4T1 murine breast cancer model, smaller tumors were observed in CD300a knockout mice compared to wild-type controls, along with a signifi-cant increase in mast cell activation. However, anti-CD300a mAb treatment did not result in a significant change in tumor weight [81].

Tumor immune evasion is facilitated, in part, by the establishment of an immunosuppressive TME, which impairs immune recognition and response. To circumvent this limitation, Gallogos and co-workers suggested three immunotherapeutic approaches targeting both inhibitory and activating pathways: (1) DSP502 a “PD-L1/PVR dual blocker” that can relieve immune suppression simultaneously by blocking both PD-L1 and its co-signaling protein PVR; (2) CD27xEGFR, a bispecific antibody targeting EGFR receptor to locally activate T cells within the TME, eliciting tumoricidal immunity while minimizing systemic toxicity; and (3) CD300a blockade, which targets an innate immune checkpoint receptor to enhance immune responses against lymphomas and uveal melanoma [96]. These methods have offered promising results in preclinical in vitro and in vivo models and may represent novel strategies in tumor immunotherapy. CD300a also inhibits mast cells, a cell type with immunomodulatory and, in some circumstances, pro-tumorigenic features in the TME. Inhibition of CD300a may therefore be expected to unleash mast cell–mediated immune responses or modify their malignant-supporting function. Blocking CD300a may have therapeutic effects similar to that of known checkpoint inhibitors and in particular targeting an immune inhibitory pathway not targeted by PD-1/PD-L1 inhibitors may further expand the current landscape of immune checkpoint strategies [97].

### 5.2. CD300 Molecules in the Regulation of TLR-Mediated Immune Signaling and Inflammation

Members of the CD300 family are key regulators of immune signaling and inflammation upon stimulation through TLRs and contribute to the maintenance of immune homeostasis. TLRs are major pattern recognition receptors through which pathogenic molecules are detected and activate immune cells and inflammation [98,99]. Accumulating evidence suggests that CD300 members control the degree of TLR activation with ensuing (Th1/Th2) orientation of the immune responses. For example, in human pDCs, TLR7 and TLR9 expression on the surface of cells is tightly linked to CD300a and CD300c expression, and TLR9-triggering upregulates CD300a expression, which might imply the existence of a negative feedback loop used to control an exaggerated immune response [57,100]. TLRs are the “alarm bells” of the immune system, whereas CD300 molecules are the “modulators” that fine tune the tone and pitch of the alarm to prevent over-activation or immune dysregulation. TLRs ring the alarm when pathogens are encountered: CD300a/c and CD300f help balance the response, thereby preventing collateral immune-mediated tissue injury.

Interaction between members of the CD300 family and TLRs is complex. Furthermore, CD300 gene transcription and protein expression could be differentially regulated in response to diverse stimuli including retinoic acid, GM-CSF, G-CSF, and IFNs [13]. Various DC subsets exhibit differential sensitivity to CD300 regulation, thereby providing several layers of diverse immune regulation. Consistent with its wide inhibitory repertoire, cross-linking human CD300a with mAbs also blocks calcium signaling induced downstream of the BCR TCR FcεRI and FcγRIIa complex [3]. Such monoclonal antibodies usually recognize the extracellular Ig-like domain of CD300a (e.g., E59. 126 mAb), that bind specifically to this region [100]. The CD300 family is able to regulate TLR-driven immune signaling cascades via multi-level mechanisms according to pathogenic stimulants, thus helping the immune system to maintain homeostasis between optimal immune response and excessive inflammation. Molecular interplay and cell-type-specific activation of CD300 molecules could be exploited for therapeutic targeting of immune activity.

## 6. Conclusions

This review summarizes the current understanding of the immunoregulatory functions of CD300a in various immune cell types, with a particular focus on its role in tumor immune evasion. CD300a, selectively expressed on myeloid and lymphoid cells, is a key inhibitory receptor that recognizes PS and PE on apoptotic or stressed cells, thereby recruiting SHP-1/2 phosphatases to the forefront for attenuation of activation signals. Tumors utilize this pathway to suppress the production of type I IFNs, killing by NK cells, and activation of mast cells and eosinophils, all contributing to dampened antitumor immunity, tumor progression, and metastasis. Hence CD300a is an innate immune checkpoint receptor with therapeutic potential to restore antitumor immunity and enhance the efficacy of adaptive checkpoint blockade.

To date, CD300a-targeted therapy remains challenging due to several unresolved questions. First, the expression and role of CD300a on different tumor-infiltrating immune cell subsets are largely unknown. For example, CD300a-mediated inhibition of DC type I IFN production is critical for T cell priming, but the nature of the tumor as well as cues from the microenvironment play a role. In addition, CD300a triggering has been linked to immune escape, and poor prognosis in certain cancers (e.g., glioblastoma [80], and diffuse large B cell lymphoma [49]), and may be stage specific in other models. Furthermore, the dual nature of type I IFN (promoting early antitumor responses along with strongly contributing to immune exhaustion upon persistent exposure) [101] suggests that the kinetics of CD300a engagement should be considered. Thus, delineating the temporal and spatial dynamics of CD300a expression across immune compartments is essential for developing effective cancer immunotherapies targeting this pathway.

Moreover, the tumor-specific mechanisms underlying CD300a-mediated immune inhibition are largely unknown. In particular, the accessory mechanisms may involve: (i) the exposure of PS or PE on tumor cells, and/or (ii) the heterogeneous expression of CD300a across immune cell subsets within the TME. However, stimulation of CD300a on its own has been established to be inhibitory for the activation of DCs, NK cells and eosinophils, the consequences of simultaneous increased CD300a engagement on multiple cell types remain relatively unexamined in vitro. Besides, the co-expression patterns of PD-1, TIM-3 and other checkpoint markers with CD300a (all in particularly within the TME- and TIM-3-expressing immunocytes) are not well characterized and these might affect therapeutic efficiency of combined immunotherapies.

We also found that how ‘strong’ or well the CD300a-binding molecule was engaged could be a critical determinant for the functional response of immune cells. This may occur especially when testing the inhibitory capacities of CD300a with antibodies cross-linked by secondary antibodies [81,93] which typically exhibit high avidity and may not fully recapitulate physiological ligand interactions, where avidity is more variable. Secondly, the binding affinity between CD300a and its ligands (PS and PE) may vary according to the type of membrane and the nature of tumor stress, thereby influencing immune evasion. Such aspects might be used in the rational design of therapeutics, as demonstrated by functional differences observed between monovalent and bivalent formats of anti-CD300a antibodies. However, since CD300a is abundantly expressed in various immune cells, it is challenging to define the contribution of CD300a as a tumor progression mediator, making it difficult to conclusively define the therapeutic benefit of CD300a blockade in cancer immunotherapy.

CD300a is a member of the immunoregulatory receptor family and, like PD-1 and CTLA-4, functions as an immune checkpoint with inhibitory properties. Its broad expression across multiple immune cell types positions it as a critical regulator of immune homeostasis. While direct clinical evidence of autoimmune toxicity following CD300a blockade is currently lacking, its structural and functional similarity to PD-1/PD-L1 suggests that abrogating its inhibitory signaling may trigger comparable immune activation and increase the risk of severe immune-related adverse events (irAEs) [102,103].

Notably, the development of irAEs has been positively correlated with improved tumor responses. Patients experiencing autoimmune toxicity often exhibit a heightened immune activation profile, such as increased CD8^+^ T cell infiltration, indicating a more robust antitumor response [104]. Accordingly, irAEs are frequently observed alongside superior therapeutic efficacy, and may surrogate biomarkers of clinical benefit, despite their associated risks [102,103]. The standard first-line management for moderate to severe irAEs typically involves systemic corticosteroids, such as prednisone or methylprednisolone [105]. Additional mitigation strategies include intermittent or reduced-dose ICI administration to preserve antitumor efficacy while limiting toxicity [106]. Furthermore, modulation of Tregs through the IL-2 pathway or low-dose cyclophosphamide has been proposed as a strategy to prevent systemic immune toxicity during checkpoint blockade-enhanced immune activation [107,108].

The therapeutic benefit of targeting CD300a has only recently begun to gain recognition, its clinical application, however, remains limited by the paucity of specific reagents and an incomplete understanding of how CD300a modulates cell-type–specific responses. Due to its wide expression on innate and adaptive immune cells, CD300a represents a compelling candidate for interventions in scenarios requiring selective modulation of immune responses or interrupting tumor-induced immunosuppression. In summary, preclinical evidence to date is exciting, but additional detailed work is required to better characterize the multifaceted effects of CD300a engagement within diverse tumor microenvironments. Such efforts will be critical to defining its therapeutic utility in future immuno-oncology strategies.
