# Correction: Peng, J.-M.; Liu, H.-Y. CD300a: An Innate Immune Checkpoint Shaping Tumor Immunity and Therapeutic Opportunity. *Cancers* 2025, *17*, 1786

**DOI:** 10.3390/cancers17233793

**Published:** 2025-11-27

**Authors:** Jei-Ming Peng, Hui-Ying Liu

**Affiliations:** 1 Institute for Translational Research in Biomedicine, Kaohsiung Chang Gung Memorial Hospital, Kaohsiung 83301, Taiwan; 2 Department of Urology, Kaohsiung Chang Gung Memorial Hospital, Graduate Institute of Clinical Medical Sciences, College of Medicine, Chang Gung University, Kaohsiung 83301, Taiwan

**Text Correction**

There was an error in the original publication. An inaccurate statement regarding the findings of study [81] was included in the original publication [1].

A correction has been made to Section 5. Therapeutic Implications, Section 5.1. CD300a Blockade as Monotherapy, Paragraph 2:

Corrected paragraph:

CD300a, a myeloid lineage-expressed inhibitory IFN-γ-induced immune checkpoint receptor with ITIMs, compromises NK cell-mediated eradication of hematologic malignancies (HMs). Therefore, CD300a is an attractive target for extending NK cell-based immunotherapy. PS was externalized on the surface of tumor cells, and CD300a bound specifically to PS. Ectopic CD300a expression in NK cells markedly reduces their cytotoxic activity on hematologic tumor cells and promotes the in vivo progression of the tumor. In contrast, the blockade of the CD300a-PS interaction by antibodies increases the transcription of cytotoxic proteins and cytokines in the NK cells, restoring their tumor-killing function. Clinical evidence also indicates that upregulated CD300a expression is associated with NK cell exhaustion and correlates with poor prognosis in patients with both hematologic and solid malignancies [93]. In the 4T1 murine breast cancer model, smaller tumors were observed in CD300a knockout mice compared to wild-type controls, along with a significant increase in mast cell activation. However, anti-CD300a mAb treatment did not result in a significant change in tumor weight [81].

The authors apologize for any inconvenience caused and state that the scientific conclusions are unaffected. This correction was approved by the Academic Editor. The original publication has also been updated.

## References

[1] Peng J.-M., Liu H.-Y. (2025). CD300a: An Innate Immune Checkpoint Shaping Tumor Immunity and Therapeutic Opportunity. Cancers.

